# Identification of Novel Anti-Inflammatory Peptides from Jellyfish *Nemopilema nomurai* Enzymatic Hydrolysate: An Integrated In Silico Analysis and Cellular Evaluation

**DOI:** 10.3390/md24060192

**Published:** 2026-05-28

**Authors:** Wen Shen, Xueqin Wang, Rongfeng Li, Song Liu, Ronge Xing, Pengcheng Li, Huahua Yu

**Affiliations:** 1 Laboratory of Experimental Marine Biology, Institute of Oceanology, Chinese Academy of Sciences, Qingdao 266000, China; 2 Laboratory for Marine Drugs and Bioproducts, Qingdao Marine Science and Technology Center, Qingdao 266237, China; 3 University of Chinese Academy of Sciences, Beijing 100049, China

**Keywords:** jellyfish, peptides, anti-inflammatory, nitric oxide, molecular docking

## Abstract

Inflammation plays a critical role in host defense and tissue repair; however, excessive or dysregulated inflammatory responses can lead to tissue damage and contribute to the progression of various diseases. Jellyfish-derived peptides have recently emerged as promising marine bioactive compounds with potential anti-inflammatory activity. In this study, three candidate anti-inflammatory peptides were identified from the enzymatic hydrolysate of *Nemopilema nomurai* through an integrated strategy combining LC–MS/MS-based peptidomics and multi-step in silico screening. The selected peptides (DGIPGMPG, PGFHVPPP, and GPKGYPGP) were prioritized based on predicted bioactivity, non-toxicity, favorable physicochemical properties, and molecular docking with the TLR4/MD-2/LPS complex (PDB ID: 3FXI), with docking scores ranging from −8.4 to −8.3 kcal/mol. Subsequent experimental validation demonstrated that all three peptides exhibited good cytocompatibility and significantly inhibited LPS-induced nitric oxide (NO) production in RAW264.7 macrophages, with GPKGYPGP showing the strongest effect. In addition, these peptides effectively reduced the secretion of pro-inflammatory cytokines, including TNF-α and IL-1β, to varying extents. Collectively, this study identifies three novel anti-inflammatory peptides derived from jellyfish enzymatic hydrolysates and highlights their potential as promising candidates for the development of marine-derived bioactive agents targeting inflammation-related diseases.

## 1. Introduction

Inflammation is a fundamental innate immune response that plays a pivotal role in host defense against pathogenic infection and tissue injury. Under physiological conditions, it acts as a protective mechanism, characterized by immune cell activation, synthesis and release of pro-inflammatory mediators (e.g., tumor necrosis factor-α (TNF-α), interleukins (IL-1, IL-6, and IL-8), and interferon-γ (IFN-γ)), and coordinated vascular responses that facilitate immune cell recruitment to damaged sites [1,2]. A moderate inflammatory response contributes to clearing harmful stimuli and restoring tissue homeostasis; however, excessive or unresolved inflammation can trigger sustained release of pro-inflammatory cytokines. This dysregulation further leads to tissue edema, pain, functional impairment, prolonged tissue damage, disrupted immune homeostasis, and impaired tissue regeneration [3]. Importantly, accumulating evidence indicates that such dysregulated inflammation is closely associated with the initiation and progression of various chronic diseases, including type 2 diabetes, inflammatory bowel disease, rheumatoid arthritis, atherosclerosis, metabolic syndrome, and inflammatory skin disorders [4,5]. Epidemiological studies have further estimated that 15–20% of malignancies are associated with chronic inflammation, highlighting its status as a major global health burden [6].

Given the profound impact of inflammatory disorders on public health, effective clinical management is critical. Currently, conventional anti-inflammatory agents, including nonsteroidal anti-inflammatory drugs (NSAIDs), glucocorticoids and immunosuppressants, remain the mainstay of treatment [7]. While these agents can alleviate inflammatory symptoms, their long-term or excessive use is accompanied by significant adverse effects. For example, NSAIDs often induce gastrointestinal complications such as dyspepsia, nausea, vomiting, abdominal pain, and heartburn [8], and are also linked to an increased risk of cardiovascular events [9]. Similarly, prolonged glucocorticoid therapy can cause immunosuppression, osteoporosis, and metabolic disturbances [10,11]. These limitations underscore an urgent need to develop safer and more effective therapeutic strategies, thereby driving growing interest in natural product-derived anti-inflammatory agents [12,13].

Among natural bioactive compounds, peptides derived from food and biological proteins have emerged as promising anti-inflammatory candidates, primarily due to their high biocompatibility and low toxicity. These peptides are typically encrypted within precursor proteins and can be released through enzymatic hydrolysis, food processing, microbial fermentation, or gastrointestinal digestion [14,15]. Mounting evidence has suggested significant anti-inflammatory activities of peptides from diverse sources, including soybeans, milk, eggs, and aquatic organisms [15]. For instance, milk-derived peptides have been reported to suppress NO production and modulate cytokine secretion in macrophages, while the casein-derived peptide QEPV specifically inhibits NO production and promotes anti-inflammatory mediator expression [16]. Similarly, the deer antler-derived peptide VH and AL significantly reduce NO production in LPS-stimulated RAW264.7 macrophages [17]. Peptides from fish and other aquatic resources also attenuate inflammatory responses by decreasing the production of NO and pro-inflammatory cytokines such as TNF-α, IL-6, and IL-1β [18]. Collectively, these findings suggest that protein-derived peptides are important natural modulators of inflammatory responses, laying the foundation for exploring novel peptide-based anti-inflammatory agents.

As a classical model for investigating inflammatory responses, lipopolysaccharide (LPS)-stimulated macrophages primarily activate signaling cascades through the Toll-like receptor 4 (TLR4)/myeloid differentiation factor 2 (MD-2) axis [19]. Upon LPS recognition, the TLR4/MD-2 complex undergoes activation, triggering downstream signaling cascades, including NF-κB and MAPK pathways [20], which ultimately lead to the production of pro-inflammatory mediators such as nitric oxide (NO) and cytokines [21]. Owing to its central role in innate immune activation, the TLR4/MD-2/LPS complex is commonly used as a structural model for investigating ligand recognition and exploring potential interactions with inflammation-related targets. Accordingly, in silico docking based on the crystal structure of the TLR4-MD-2-LPS complex (PDB ID: 3FXI) has been commonly employed to explore potential ligand–receptor interactions and provide preliminary structural insights into interaction patterns within the TLR4 signaling complex [22].

Marine organisms represent a rich reservoir of structurally diverse bioactive compounds, and jellyfish have recently attracted increasing attention due to their abundant biomass, high protein content, and underexploited utilization potential. As gelatinous marine invertebrates, jellyfish *Nemopilema nomurai* (*N. nomurai*) is widely distributed in the coastal areas of East Asia and serves as a dominant species in jellyfish blooms, thereby making it an abundant and accessible marine resource [23,24]. Their dry matter is rich in proteins, collagen and polysaccharides, rendering them promising raw materials for applications in food, biomaterials, and biomedicine [25,26,27]. Importantly, the high protein content of jellyfish provides a favorable basis for producing functional peptide via enzymatic hydrolysis. Previous studies have demonstrated that jellyfish-derived proteins, collagen, and peptide hydrolysates possess multiple biological activities, including antioxidant, anti-inflammatory, immunomodulatory, and cytoprotective effects [28,29,30,31,32]. For example, proteins from *Rhizostoma pulmo* and their low-molecular-weight hydrolysates exhibited antioxidant activity and protected keratinocytes from oxidative stress [28], jellyfish collagen hydrolysates suppressed the expression of inflammatory mediators (e.g., TNF-α, IL-1β, and IL-8) in animal models [29], and jellyfish-derived peptide showed strong radical-scavenging and immunomodulatory activities, with low-molecular-weight peptides identified as the key functional components [30]. Additional studies have reported antihypertensive effects of collagen peptides from *Rhopilema esculentum* in animal models [31], and protective effects of peptide JPHT-2 on keratinocytes against oxidative damage via enhancing intracellular antioxidant defenses [32]. These findings collectively suggest that jellyfish-derived peptides are promising bioactive molecules with great potential for biomedical applications, particularly in anti-inflammatory therapy.

Despite these advances, most studies have primarily focused on the overall bioactivities of jellyfish hydrolysates, whereas the systematic identification and experimental validation of specific bioactive peptides remain limited. Given the complex composition of enzymatic hydrolysates, it is increasingly important to establish strategies that can efficiently screen and validate functional peptide components responsible for the observed biological activities. To address this gap, integrated peptidomics and bioinformatic approaches have been widely applied to identify candidate peptides from complex mixtures and to link peptide sequences with specific bioactivities. In this study, we focused on the discovery and validation of anti-inflammatory peptides from jellyfish-derived hydrolysates. Peptide sequences identified by LC–MS/MS were subjected to multi-step in silico screening and molecular docking to prioritize candidates with potential anti-inflammatory activity. Three candidate peptides were subsequently selected, chemically synthesized, and experimentally validated using LPS-stimulated RAW264.7 macrophages. By combining computational prediction with cellular validation, this study provides a targeted strategy for identifying functional anti-inflammatory peptides from complex marine-derived peptide mixtures and offers new insights into their potential application in inflammation-related disorders.

## 2. Results

### 2.1. JP-FC Showed No Apparent Cytotoxicity Toward RAW264.7 Cells

**Figure 1:**
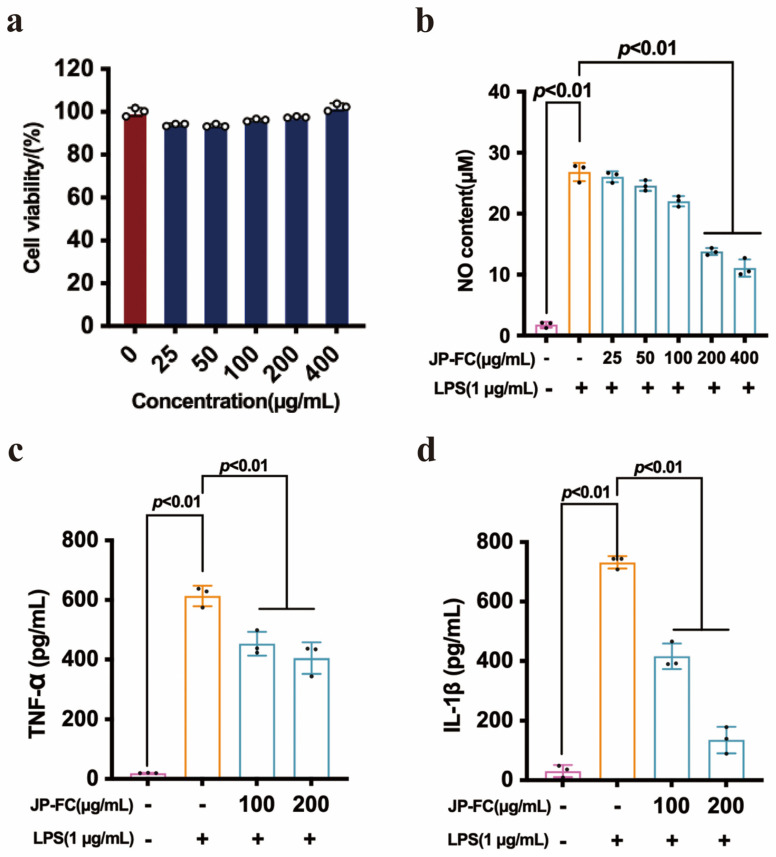
(**a**) Effect of JP-FC on the viability of RAW264.7 cells, the red bar represents the blank control group, and the blue bars represent the different concentration groups of JP-FC. (**b**) Concentration-dependent inhibitory effects of JP-FC on LPS-induced NO production in RAW264.7 macrophages. (**c**) Effect of JP-FC on the production of pro-inflammatory cytokines TNF-α and (**d**) IL-1β in LPS-stimulated RAW264.7 cells. In panels (**b**–**d**), the pink bar represents the blank control group, the yellow bar represents the LPS-stimulated model group, and the blue bars represent the groups treated with LPS and different concentrations of JP-FC. Data are presented as mean ± SD (*n* = 3). Statistical significance was indicated as *p* < 0.01.

Prior to evaluating its anti-inflammatory activity, the cytotoxicity of JP-FC was assessed in RAW264.7 macrophages. As shown in Figure 1a, JP-FC did not exhibit any significant inhibitory effect on cell viability across the tested concentration range (25–400 μg/mL). Cell viability remained above 90% in all treatment groups, with no statistically significant differences compared to the control group. Notably, a slight increase in cell viability was observed at 200 and 400 μg/mL, suggesting a potential proliferative or metabolic enhancement effect, although no cytotoxicity was detected. These findings indicate that JP-FC possesses good cytocompatibility under the experimental conditions, supporting its suitability for subsequent anti-inflammatory evaluation.

### 2.2. JP-FC Attenuated LPS-Induced Inflammatory Responses in RAW264.7 Cells

LPS stimulation (1 μg/mL, 6 h) significantly increased nitric oxide (NO) production and the secretion of pro-inflammatory cytokines TNF-α and IL-1β in RAW264.7 macrophages, suggesting successful establishment of the inflammatory model. JP-FC treatment reduced NO production in a concentration-dependent manner, with the high-dose group exhibiting a stronger inhibitory effect on LPS-induced inflammatory responses (Figure 1b). To further characterize its anti-inflammatory activity, additional concentration gradients (25–400 μg/mL) were subsequently evaluated, and the corresponding NO inhibition rates and apparent IC_50_ value are provided in the Supplementary Materials (Figure S1). In addition, JP-FC significantly decreased the secretion of TNF-α and IL-1β at 100 and 200 μg/mL (Figure 1c,d). At 200 μg/mL, TNF-α levels were reduced from 613.80 ± 34.29 pg/mL in the model group to 405.08 ± 52.56 pg/mL, while IL-1β decreased from 732.12 ± 20.74 pg/mL to 136.06 ± 44.62 pg/mL. These results indicate that JP-FC effectively suppresses LPS-induced inflammatory responses by inhibiting NO overproduction and downregulating pro-inflammatory cytokine secretion.

### 2.3. GO and KEGG Enrichment Analysis of Precursor Proteins Identified from Peptide Sequences

**Figure 2:**
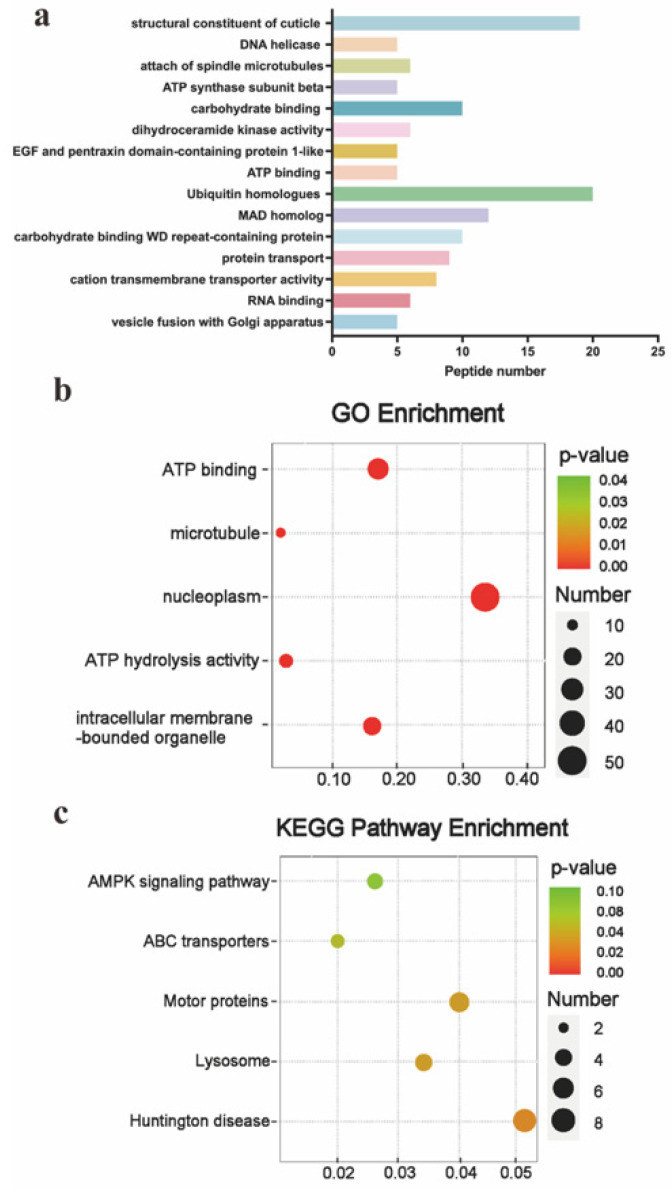
(**a**) Distribution of the top 15 precursor proteins ranked by peptide identification scores. The bar colors are used solely for visual distinction among the listed functional categories and have no additional biological or statistical meaning. (**b**) GO enrichment analysis of precursor proteins identified from JP-FC-derived peptide sequences, showing the top five enriched GO terms. (**c**) KEGG pathway enrichment analysis of precursor proteins, displaying the top five enriched pathways.

Peptide profiling of JP-FC was performed using LC-MS/MS, leading to the identification of 2221 high-confidence peptide sequences. These peptides were mapped to 1427 precursor proteins based on the UniProtKB database (phylum Cnidaria, taxon ID: 332258). The top 15 precursor proteins ranked by confidence scores were functionally annotated, including structural constituent of cuticle, DNA helicase, spindle microtubule attachment, ATP synthase subunit beta, carbohydrate binding proteins, dihydroceramide kinase activity, EGF- and pentraxin domain-containing protein 1-like, ATP binding proteins, ubiquitin homologues, MAD homolog, WD repeat-containing proteins, protein transport-related proteins, cation transmembrane transporter activity, RNA binding proteins, and vesicle fusion with the Golgi apparatus (Figure 2a). To further explore the functional characteristics of these precursor proteins from a systems biology perspective, Gene Ontology (GO) and Kyoto Encyclopedia of Genes and Genomes (KEGG) enrichment analyses were performed. Although the biological activities of short peptides are not necessarily directly inherited from their precursor proteins, annotation of the parent proteins can provide valuable insights into the functional background of the peptide pool and their potential involvement in biological processes. The top five enriched GO terms and KEGG pathways were selected for visualization (Figure 2b). GO analysis indicated that the precursor proteins were primarily associated with structural organization, energy metabolism, signal transduction, and molecular transport processes. KEGG pathway enrichment analysis revealed that the top five pathways included Huntington’s disease, motor proteins, and ABC transporters (Figure 2c). Notably, pathways such as lysosome and AMPK signaling are closely associated with inflammatory regulation and cellular stress responses.

### 2.4. Multi-Step Bioinformatic Screening Identified Three Candidate Anti-Inflammatory Peptides

To identify potential anti-inflammatory peptides from JP-FC, a multi-step bioinformatic screening workflow was implemented. Peptides with PeptideRanker scores > 0.8 (http://distilldeep.ucd.ie/PeptideRanker/, accessed on 15 May 2026) were first retained to minimize false-positive predictions, and only those predicted to be non-toxic by ToxinPred (http://crdd.osdd.net/raghava/toxinpred/, accessed on 15 May 2026) were selected. Subsequent filtering based on cell-penetrating potential using CPPpred (http://bioware.ucd.ie/~compass/biowareweb/Server_pages/cpppred.php, accessed on 15 May 2026), anti-inflammatory prediction scores from CSM-peptides (https://biosig.lab.uq.edu.au/csm_peptides/predict, accessed on 15 May 2026), and novelty further refined the candidate pool.

**Figure 3:**
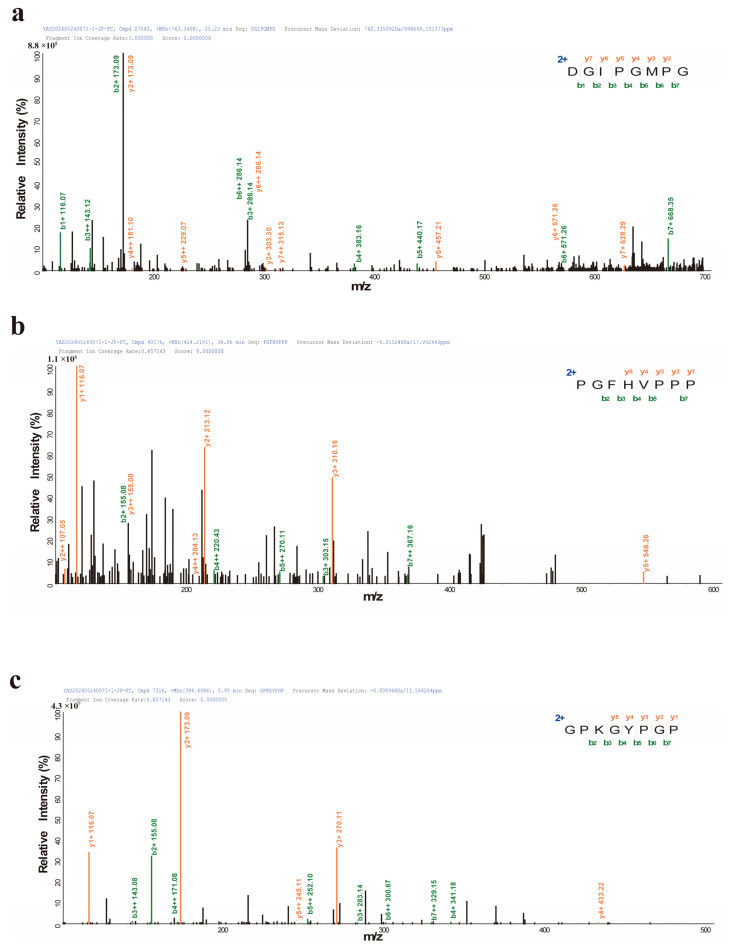
The MS/MS spectra of the identified peptides identified from JP-FC. (**a**) DGIPGMPG, (**b**) PGFHVPPP, (**c**) GPKGYPGP. Fragment ions corresponding to b- and y-type ions are indicated. The green and orange annotations indicate b-type and y-type fragment ions, respectively. The b- and y-ion series correspond to peptide fragments generated from the N- and C-termini during MS/MS fragmentation, respectively, supporting the sequence assignment of the identified peptides.

As summarized in Table 1, three peptides, DGIPGMPG, PGFHVPPP, and GPKGYPGP, were ultimately selected as candidate anti-inflammatory peptides. All three peptides were predicted to be non-toxic and exhibited favorable physicochemical and bioavailability-related properties. Their anti-inflammatory scores predicted by CSM-peptides ranged from 0.54 to 0.71, suggesting potential anti-inflammatory activity. Furthermore, BIOPEP-UWM analysis (https://biochemia.uwm.edu.pl/biopep/start_biopep.php, accessed on 15 May 2026) indicated that these peptides have not been previously reported with suggested anti-inflammatory activity, supporting their novelty as functional candidates (Table 1). Collectively, this multi-criteria screening strategy enabled the identification of three promising candidate peptides for subsequent experimental validation. The sequences of DGIPGMPG, PGFHVPPP, and GPKGYPGP were further suggested by LC-MS/MS analysis, and the corresponding MS/MS fragmentation spectra are shown in Figure 3.

### 2.5. Molecular Docking Suggested Stable Interactions Between Candidate Peptides and the TLR4/MD-2/LPS Complex

**Figure 4:**
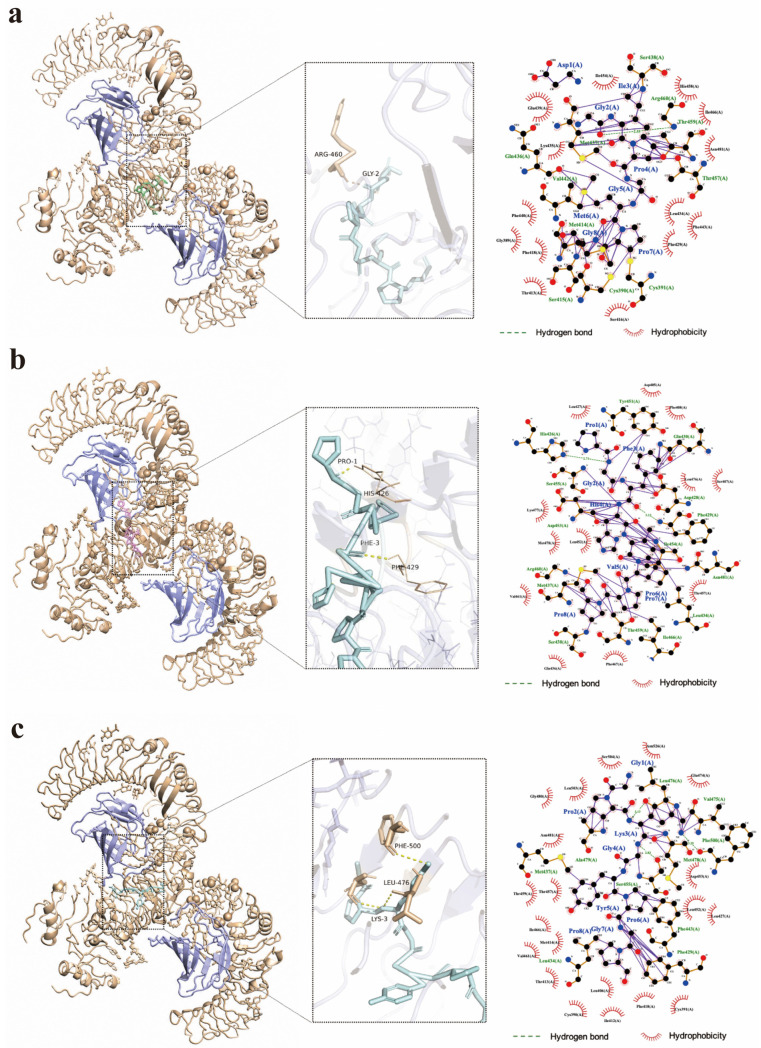
Predicted 3D and 2D molecular interactions between the identified anti-inflammatory peptides and the TLR4/MD-2/LPS complex (PDB ID: 3FXI). In the 3D interaction models, TLR4 is shown in orange and MD-2 is shown in purple to clearly distinguish the components of the receptor complex. The peptide ligands are displayed within the binding region of the complex. (**a**) DGIPGMPG, (**b**) PGFHVPPP, and (**c**) GPKGYPGP. The 3D representations illustrate the spatial orientation of each peptide within the docking region, while the corresponding 2D interaction diagrams depict residue-level interactions between the peptides and the receptor complex. In the 2D diagrams, peptide ligand bonds are shown in purple, non-ligand/receptor bonds are shown in orange/brown, hydrogen bonds are indicated by green dashed lines with bond lengths labeled in Å, and hydrophobic contacts are represented by red semicircular arcs surrounding the interacting residues. Atoms are colored according to element type, with carbon shown in black, oxygen in red, and nitrogen in blue. The dashed boxes indicate enlarged or emphasized regions used to improve the visibility of local interaction details, particularly where residue labels or interaction lines are close to each other. Although some labels and interaction elements partially overlap due to the spatial proximity of residues in the 2D projection, the key interaction information, including hydrogen bonds, hydrophobic contacts, interacting residues, and ligand/receptor connectivity, remains interpretable. These interaction patterns suggest potential interactions of the peptides with residues located near the TLR4/MD-2 interface region.

To explore potential structural correlates underlying their anti-inflammatory activity, DGIPGMPG, PGFHVPPP, and GPKGYPGP were docked to the human LPS-bound TLR4/MD-2 complex (PDB ID: 3FXI). The predicted binding energies were similar among the three peptides (−8.4 to −8.3 kcal/mol), indicating comparable binding affinities under the selected docking conditions (Table 1). The top-ranked docking poses revealed potential polar interactions, including hydrogen bonds between DGIPGMPG and Arg460/Gly2, PGFHVPPP and His426/Phe429, and GPKGYPGP and Leu476/Lys3 (Figure 4, Table 2). In addition, all peptides formed multiple hydrophobic interactions with residues surrounding the predicted binding region, suggesting favorable surface complementarity. It should be noted that, since 3FXI represents an LPS-bound receptor complex, these docking results are primarily hypothesis-generating and reflect potential binding compatibility rather than direct evidence of LPS displacement or definitive inhibition of TLR4 signaling. Nevertheless, the observed interaction patterns provide a structural basis for further investigation into whether these peptides can modulate TLR4/MD-2-mediated inflammatory responses in subsequent target validation and pathway-specific studies.

### 2.6. Synthetic Candidate Peptides Exhibited Good Cytocompatibility in RAW264.7 Cells

**Figure 5:**
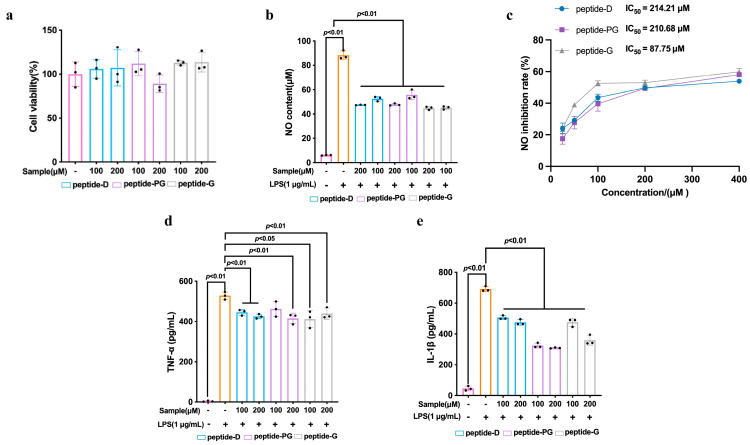
Effects of synthetic anti-inflammatory peptides DGIPGMPG (peptide-D), PGFHVPPP (peptide-PG), and GPKGYPGP (peptide-G) on RAW264.7 cells. (**a**) Cell viability. (**b**) NO production and (**c**) concentration-dependent inhibition of LPS-induced NO production in RAW264.7 cells treated with peptide-D, peptide-PG, and peptide-G (25–400 μM). (**d**) Secretion of pro-inflammatory cytokines TNF-α and (**e**) IL-1β in LPS-stimulated RAW264.7 cells. Data are presented as mean ± SD of three independent experiments (*n* = 3). Statistical significance was indicated as *p* < 0.01 and *p* < 0.05.

The cytocompatibility of the three candidate peptides was evaluated prior to anti-inflammatory assessment. As shown in Figure 5a, DGIPGMPG (peptide-D), PGFHVPPP (peptide-PG), and GPKGYPGP (peptide-G) did not exhibit any significant cytotoxic effects on RAW264.7 cells at either 100 or 200 μM. Specifically, DGIPGMPG maintained cell viabilities of 105.90 ± 10.70% and 107.23 ± 20.62% at 100 and 200 μM, respectively. PGFHVPPP showed viabilities ranging from 89.05 ± 10.15% to 111.87 ± 13.68%, while GPKGYPGP exhibited slightly higher values, ranging from 112.90 ± 2.71% to 113.61 ± 11.19% (Figure 5a). No statistically significant differences were observed compared with the control group. Values slightly exceeding 100% may reflect enhanced cellular metabolic activity rather than increased cell number. These results demonstrate that all three peptides possess good cytocompatibility under the tested conditions, supporting their suitability for subsequent anti-inflammatory evaluation.

### 2.7. Candidate Peptides Attenuated LPS-Induced Inflammatory Responses in RAW264.7 Cells

To further evaluate the concentration-dependent anti-inflammatory activity of JP-FC and the identified peptides, additional concentration gradients were analyzed and apparent IC_50_ values based on NO inhibition were estimated. The results demonstrated concentration-dependent inhibitory effects on LPS-induced NO production. Among the three peptides, peptide-G exhibited the strongest inhibitory activity with an apparent IC_50_ value of 87.75 μM, followed by peptide-PG (210.68 μM) and peptide-D (214.21 μM), indicating differences in their relative anti-inflammatory potency (Figure 5c). All three peptides significantly suppressed nitric oxide (NO) overproduction in LPS-stimulated RAW264.7 cells (Figure 5b). Peptide-D exhibited inhibition rates of 43.49 ± 2.24% and 49.75 ± 0.25% at 100 and 200 μM, respectively, while peptide-PG showed inhibition rates of 39.66 ± 4.68% and 49.35 ± 1.16%. Consistent with the NO results, ELISA analysis demonstrated that all three peptides significantly reduced the secretion of pro-inflammatory cytokines TNF-α and IL-1β in LPS-stimulated cells (Figure 5d,e). Among them, peptide-PG exhibited a relatively stronger inhibitory effect. Collectively, these findings indicate that the selected peptides effectively attenuate macrophage inflammatory activation by suppressing NO production and downregulating pro-inflammatory cytokine secretion. Moreover, these results validate the reliability of the multi-step screening strategy and support the functional relevance of the identified peptides.

## 3. Discussion

The present study demonstrates that JP-FC exerts anti-inflammatory effects in LPS-stimulated RAW264.7 macrophages, as evidenced by reduced NO production and decreased secretion of TNF-α and IL-1β. To explore potential structural determinants underlying the activity of DGIPGMPG, PGFHVPPP, and GPKGYPGP, sequence-based feature analysis was integrated with molecular docking. These computational results are interpreted as hypothesis-generating evidence to support candidate prioritization, rather than definitive mechanistic proof. These findings extend the current understanding of jellyfish-derived peptides as functional modulators of macrophage-mediated inflammatory responses.

### 3.1. Molecular Weight and Potential Target Engagement

DGIPGMPG, PGFHVPPP, and GPKGYPGP fall within a narrow low-molecular-weight range (742.84–847.09 Da). Short peptides are generally considered advantageous in discovery workflows, as reduced steric hindrance and greater conformational flexibility may facilitate interactions with protein surfaces [33,34]. Docking to the human TLR4/MD-2/LPS complex (PDB: 3FXI) yielded binding energies below −8.0 kcal/mol for all three peptides within the selected docking region, suggesting that energetically favorable binding poses are feasible in silico [35]. TLR4 is a key pattern recognition receptor mediating LPS-induced inflammatory responses in macrophages and has been extensively investigated as a molecular target in anti-inflammatory studies. As the LPS-induced production of NO and pro-inflammatory cytokines is primarily mediated by TLR4-dependent signaling pathways, the inhibitory effects observed for these peptides may be associated with potential modulation of TLR4 activation. Notably, the TLR4/MD-2 axis is widely recognized as a central mediator of inflammatory signaling, and a range of inhibitors targeting this complex have been reported [36]. However, it is important to note that docking scores do not directly correspond to binding affinity or inhibitory potency. Therefore, these results should be interpreted as providing structural plausibility for potential target engagement, rather than direct evidence of TLR4 pathway modulation.

### 3.2. Contributions of Residue Composition and Local Interactions

Peptide physicochemical properties, including charge distribution, hydrophobicity, aromatic residues, and conformational preferences, are known to influence peptide–protein interactions and functional outcomes [33,37]. In general, basic residues (e.g., Lys and Arg) may facilitate electrostatic interactions with negatively charged or polar regions, while moderate hydrophobicity can enhance compatibility with hydrophobic interfaces. Aromatic residues may further stabilize interactions through hydrophobic packing and π-related interactions [37]. Among the identified peptides, DGIPGMPG contains an acidic residue (Asp), whereas GPKGYPGP includes a basic residue (Lys), contributing to localized charge diversity. Notably, ligand recognition within the TLR4/MD-2/LPS complex involves multiple charged regions, and electrostatic interactions between LPS phosphate groups and positively charged residues are known to stabilize ligand binding [35]. Consistent with this context, docking suggested that DGIPGMPG may form a hydrogen bond with Arg460, while GPKGYPGP exhibited a polar interaction involving Lys3, potentially contributing to pose stabilization.

However, the presence of charged residues alone is unlikely to determine anti-inflammatory activity. Their positional context (N-terminal, C-terminal, or internal) and the overall physicochemical profile of the peptide must also be considered. Indeed, previous studies have reported anti-inflammatory peptides containing acidic residues (e.g., pEL, γ-EC, and γ-EV), indicating that activity cannot be explained by charge alone [33,38]. Similarly, peptides with C-terminal Arg residues (e.g., PRRTRMMNGGR and GPR) have demonstrated anti-inflammatory activity in LPS-stimulated macrophage models, suggesting that terminal basic residues may be compatible with, but not determinative of, bioactivity [39,40].

In addition to electrostatic interactions, hydrophobic effects may also contribute to peptide-receptor recognition. The TLR4/MD-2 complex contains a pronounced hydrophobic pocket within MD-2 that accommodates LPS [35]. PGFHVPPP is enriched in Pro and Phe, GPKGYPGP contains Tyr, and DGIPGMPG includes hydrophobic residues such as Ile and Met. Docking suggested multiple hydrophobic interactions between these peptides and residues within the selected docking region, which may contribute to favorable binding poses. Aromatic residues in PGFHVPPP (Phe) and GPKGYPGP (Tyr) may further stabilize interactions through π-related effects, consistent with observed contacts involving aromatic residues such as Phe418 and Phe429 in the 3FXI structure [41]. Taken together, the observed anti-inflammatory effects in LPS-stimulated RAW264.7 macrophages, combined with docking results, suggest that DGIPGMPG, PGFHVPPP, and GPKGYPGP possess physicochemical features compatible with potential interactions at the TLR4/MD-2/LPS interface. Nevertheless, these findings provide structural hypotheses rather than mechanistic confirmation. Future studies incorporating pathway-specific assays (e.g., TLR4/NF-κB reporter assays, phosphorylation analysis of downstream signaling proteins, or direct binding measurements) will be required to elucidate whether and how these peptides modulate TLR4/MD-2-mediated inflammatory signaling. In addition, it should be noted that the present study focuses on the identification and validation of a limited number of candidate anti-inflammatory peptides from a complex enzymatic hydrolysate, rather than providing a comprehensive functional evaluation of all peptide components. Enzymatic hydrolysates typically contain a large number of peptide sequences with diverse physicochemical properties and potential bioactivities, making it impractical to experimentally assess each individual peptide. To address this challenge, an integrated screening strategy combining LC–MS/MS-based peptidomics with multi-step in silico analysis was employed to prioritize candidate peptides with a higher likelihood of bioactivity. In particular, the CSM-peptides platform, which is based on machine learning models incorporating sequence-derived and physicochemical descriptors, has been previously developed and benchmarked against other prediction approaches and reported to provide predictive capability of peptide bioactivity, including anti-inflammatory potential [42].

In this context, peptides with lower prediction scores or unfavorable properties were not included as experimental controls, as the primary objective was to validate the biological activity of prioritized candidates rather than to perform a full comparative analysis across all peptide sequences. This targeted validation strategy has been commonly employed in recent studies on bioactive peptides derived from complex natural sources.

Nevertheless, it should be acknowledged that molecular docking and bioinformatic prediction provide only preliminary, hypothesis-generating insights into potential peptide–target interactions. In addition, docking scores alone may not directly predict cellular anti-inflammatory potency, since peptide activity in biological systems is influenced by multiple factors beyond static receptor–ligand interaction estimates, including structural flexibility, stability, membrane interaction, and intracellular accessibility. Although docking analysis and cytokine inhibition results suggest possible relevance to TLR4-associated inflammatory signaling, direct pathway validation was not performed in the present study. Therefore, additional mechanistic studies, including analysis of TLR4/NF-κB/MAPK-related signaling events, will be necessary to clarify whether and how these peptides influence inflammation-related signaling processes.

In addition, future studies incorporating additional control peptides and comparative experimental validation will further strengthen the understanding of peptide specificity and mechanisms of action.

## 4. Materials and Methods

### 4.1. Materials

N-acetyl-L-cysteine, lipopolysaccharide (LPS), N-(1-naphthyl) ethylenediamine dihydrochloride, and ELISA kits for tumor necrosis factor-α (TNF-α) and interleukin-1β (IL-1β) were purchased from Solarbio Science & Technology Co., Ltd. (Beijing, China). Fetal bovine serum (FBS) and DMEM culture medium were obtained from HyClone (Logan, UT, USA). RAW264.7 cells were obtained from the China Infrastructure of Cell Line Resources (Beijing, China). All other chemicals used were of analytical or HPLC grade.

### 4.2. Preparation of JP-FC

The JP-FC hydrolysate was prepared according to our previously reported method with slight modifications [32,43]. Briefly, fresh umbrella tissues of *N. nomurai* were washed with deionized water, homogenized, lyophilized, and then dispersed in deionized water at a solid-to-liquid ratio of 1:40 (*w*/*v*). Sequential enzymatic hydrolysis was conducted using flavourzyme and compound protease. The first hydrolysis step was performed with flavourzyme at an enzyme-to-substrate ratio of 3000 U/g at 50 °C for 10 h, followed by a second hydrolysis with compound protease at 4000 U/g under the same temperature and duration. The reaction was terminated by heating at 100 °C for 10 min. After cooling, the hydrolysate was centrifuged at 18,000× *g* for 15 min at 4 °C, and the supernatant was collected and lyophilized to obtain JP-FC. The basic composition and molecular-weight distribution of JP-FC were consistent with our previous characterization: the protein and peptide contents were 71.70 and 54.51 g/100 g DW, respectively, and the fraction below 1000 Da accounted for 37.61% of the total hydrolysate. 

### 4.3. Cell Culture

RAW264.7 murine macrophages were cultured according to the method described by Li et al. [44]. Cells were maintained in high-glucose DMEM supplemented with 10% FBS under humidified conditions at 37 °C with 5% CO_2_. The culture medium was refreshed every 1–2 days, and cells were subcultured when reaching 80–90% confluence.

### 4.4. Cell Viability

Cell viability was evaluated using the MTT assay as previously described by Teng et al. [32] with minor modifications. RAW264.7 cells were seeded into 96-well plates at a density of 1 × 10^4^ cells per well and incubated for 24 h. Subsequently, cells were treated with JP-FC at final concentrations of 100, 200, and 400 μg/mL for an additional 24 h. Prior to treatment, JP-FC solutions were sterilized using a 0.22 μm membrane filter. Following incubation, cell viability was determined by the MTT assay. Wells containing untreated cells served as the control group, while wells without cells were used as blanks. Absorbance was measured at 490 nm using a microplate reader (Thermo Fisher Scientific, Waltham, MA, USA). Cell viability was calculated according to the following equation:

$$\mathrm{Cell}\;\mathrm{viability}\;(\%)=\frac{A_{490\;\mathrm{nm},\;\mathrm{sample}}\;-\;A_{490\;\mathrm{nm},\;\mathrm{blank}}}{A_{490\;\mathrm{nm},\;\mathrm{control}}\;-\;A_{490\;\mathrm{nm},\;\mathrm{blank}}}\;\times \;100$$

where A_sample_, A_control_, and A_blank_ represent the absorbance of the sample-treated group, untreated control group, and blank group (medium without cells), respectively.

### 4.5. LPS-Induced Inflammatory Model and JP-FC Treatment

RAW264.7 cells were seeded in 96-well plates and allowed to adhere for 24 h. Cells were pretreated with JP-FC at concentrations of 100 and 200 μg/mL for 24 h, followed by stimulation with lipopolysaccharide (LPS, 1 μg/mL) for 6 h to establish an inflammatory model. Cells without any treatment served as the blank control group, while cells treated with LPS alone were used as the model group.

### 4.6. Determination of NO Production

NO production was assessed by measuring nitrite levels in the culture supernatants using the Griess reaction. Briefly, 50 μL of cell culture supernatant was mixed with 50 μL of Griess reagent I and incubated for 5 min at room temperature, followed by the addition of 50 μL of Griess reagent II and further incubation for 5–10 min in the dark. Absorbance was measured at 540 nm using a microplate reader. NO concentration was calculated based on a sodium nitrite (NaNO_2_) standard curve and expressed as μmol/L.

### 4.7. Determination of TNF-α and IL-1β

RAW264.7 cells were seeded in 6-well plates and incubated for 24 h. Cells were pretreated with JP-FC at concentrations of 100 and 200 μg/mL for 24 h, followed by stimulation with lipopolysaccharide (LPS, 1 μg/mL) for 6 h to establish an inflammatory model. After treatment, culture supernatants were collected and centrifuged to remove cellular debris, and subsequently stored at −80 °C until analysis. The levels of TNF-α and IL-1β were quantified using ELISA kits according to the manufacturers’ protocols.

### 4.8. LC-MS/MS Analysis

Peptide identification was performed using a Q Exactive mass spectrometer (Thermo Fisher Scientific, Waltham, MA, USA) coupled with an EASY-nLC system. Peptides were separated on a C18 column with a linear gradient using 0.1% formic acid in water (mobile phase A) and 0.1% formic acid in acetonitrile (mobile phase B). MS data were acquired in data-dependent acquisition mode and processed against a UniProtKB Cnidaria database (taxonomy ID: 332258). Carbamidomethylation of cysteine was set as a fixed modification and methionine oxidation as a variable modification. Only high-confidence peptide identifications were retained for further analysis. Peptides were filtered at a false discovery rate (FDR) < 1%. The base peak chromatogram (BPC) of JP-FC obtained by LC-MS/MS analysis for peptide profiling is shown in Supplementary Materials (Figure S2).

### 4.9. In Silico Analysis of Anti-Inflammatory Peptides in JP-FC

The potential bioactivity of peptide sequences identified from JP-FC was initially evaluated using the PeptideRanker online server (http://distilldeep.ucd.ie/PeptideRanker/, accessed on 15 May 2026), which predicts the likelihood of a peptide being bioactive based on a machine learning model trained on known functional and non-functional peptides [45]. The output score ranges from 0 to 1, with higher scores indicating a greater probability of biological activity. Peptides with scores greater than 0.8 were selected to reduce false-positive predictions. Peptide toxicity was assessed using ToxinPred (http://crdd.osdd.net/raghava/toxinpred/, accessed on 15 May 2026), a support vector machine (SVM)-based tool that predicts peptide toxicity based on sequence features, physicochemical properties, and known toxic peptide datasets [46]. Only peptides predicted to be non-toxic were retained for further analysis. Cell-penetrating potential was evaluated using CPPpred (http://bioware.ucd.ie/~compass/biowareweb/Server_pages/cpppred.php, accessed on 15 May 2026), which applies a machine learning-based classifier to estimate the likelihood of peptides entering cells based on sequence-derived descriptors, as intracellular accessibility is considered an important factor for biological activity [47]. The potential anti-inflammatory activity of the selected peptides was predicted using the CSM-peptides server (https://biosig.lab.uq.edu.au/csm_peptides/predict, accessed on 15 May 2026), which employs a machine learning framework integrating graph-based structural signatures and sequence-derived features, including physicochemical properties, evolutionary information, and predicted secondary structure, to estimate peptide bioactivity [42]. Higher prediction scores indicate a higher probability of anti-inflammatory activity. Peptide novelty was assessed using the BIOPEP-UWM database (https://biochemia.uwm.edu.pl/biopep/start_biopep.php, accessed on 15 May 2026), which contains curated information on known bioactive peptides [48]. Sequences without previously reported anti-inflammatory activity were considered novel candidates.

Based on an integrated evaluation of predicted bioactivity, toxicity, cell-penetrating potential, physicochemical characteristics (e.g., hydrophobicity and net charge), and novelty, peptides with relatively high predicted activity and acceptable safety profiles were selected for subsequent experimental validation.

### 4.10. Molecular Docking of Potential Anti-Inflammatory Peptides Identified from JP-FC

Molecular docking was performed to explore the potential interactions between selected peptides and inflammation-related molecular targets. The crystal structure of the human TLR4/MD-2/LPS complex (PDB ID: 3FXI) was retrieved from the RCSB Data Bank. Structure preparation was performed using PyMOL 2.4, including removal of water molecules and the addition of polar hydrogen atoms, while the co-crystallized LPS molecule was retained to preserve the native receptor–ligand complex. Three-dimensional structures of candidate peptides were constructed based on their amino acid sequences using ChemDraw 19.0 and Chem3D 19.0. The lowest-energy conformations were selected and saved in MOL format, followed by conversion into PDB format using OpenBabel 2.3.2. Docking simulations were performed using AutoDock 4.2 with the Lamarckian genetic algorithm under default parameters. The grid box was defined to cover the receptor-binding interface based on reported active sites. Each docking simulation was repeated 10 times, and the conformation with the lowest binding energy was selected as the optimal binding mode. Protein–peptide interactions, including hydrogen bonding and hydrophobic interactions, were analyzed and visualized using PyMOL 2.4 and LigPlot 2.2.9.

### 4.11. Synthesis of Peptides

The screened novel peptides, DGIPGMPG (peptide-D), PGFHVPPP (peptide-PG), and GPKGYPGP (peptide-G), were synthesized by TGpeptide Biotechnology Co., Ltd. (Nanjing, China) using solid-phase peptide synthesis. The purity of the synthesized peptides (>98%) was confirmed by HPLC and electrospray ionization mass spectrometry (ESI-MS). The HPLC purity profiles and mass spectrometry characterization of the three peptides are shown in Supplementary Materials (Figures S3–S5), and the corresponding HPLC purity analysis is summarized in Supplementary Table S1.

### 4.12. Cell Viability of Synthetic Peptides

RAW264.7 cells were treated with DGIPGMPG, PGFHVPPP, and GPKGYPGP at final concentrations of 100 and 200 μM for 24 h. Cell viability was assessed using the MTT assay, and absorbance was measured at 490 nm using a microplate reader.

### 4.13. Evaluation of Anti-Inflammatory Activity of Synthetic Peptides

RAW264.7 cells were stimulated with LPS (1 μg/mL) for 6 h, followed by treatment with DGIPGMPG, PGFHVPPP, and GPKGYPGP at concentrations of 100 and 200 μM for 24 h. NO production in the culture supernatants was determined using the Griess assay. The levels of TNF-α and IL-1β were measured using ELISA kits according to the manufacturers’ protocols.

### 4.14. Statistical Analysis

All data are presented as mean ± standard deviation (SD) from three independent biological replicates (*n* = 3). Statistical analyses were performed using GraphPad Prism 9.5.1. Comparisons among multiple groups were conducted using one-way analysis of variance (ANOVA) followed by appropriate multiple comparison tests. Comparisons between two groups were performed using an unpaired two-tailed Student’s *t*-test. Differences were considered statistically significant at *p* < 0.05 and highly significant at *p* < 0.01.

## 5. Conclusions

In this study, three novel anti-inflammatory peptides, DGIPGMPG, PGFHVPPP, and GPKGYPGP, were identified from a jellyfish enzymatic hydrolysate (JP-FC) from *N. nomurai* by integrating LC-MS/MS-based peptidomics with multi-step bioinformatic screening and molecular docking. These peptides exhibited favorable physicochemical properties, non-toxicity, and predicted anti-inflammatory potential. Subsequent experimental validation suggested that all three peptides effectively suppressed inflammatory mediator production in macrophages, with GPKGYPGP showing the most pronounced activity. Structural analysis suggested that low molecular weight, charge distribution, hydrophobic residues, and aromatic amino acids may collectively contribute to the observed bioactivity. Molecular docking further provided hypothesis-generating insights into potential interactions with the TLR4/MD-2/LPS complex, supporting their possible involvement in inflammation-related signaling pathways.

Overall, this study establishes a link between the bioactivity of JP-FC and its functional peptide components, highlighting jellyfish-derived peptides as a promising source of novel anti-inflammatory agents. These findings provide a scientific basis for the development and application of marine-derived bioactive peptides in the fields related to biomedicine and functional foods.

Nevertheless, further studies are required to deepen the understanding of the underlying mechanisms. In particular, future work should focus on elucidating the direct interactions between active peptides and key molecular targets involved in inflammatory signaling pathways. In addition, direct experimental validation of pathway-specific signaling events was not performed in the present study, and potential assay interference or endotoxin-related effects cannot be completely excluded. Therefore, additional mechanistic studies and experimental controls will be necessary to further strengthen the interpretation of the observed anti-inflammatory activity.

In addition, in vivo validation and pharmacokinetic evaluation are necessary to assess their efficacy, stability, and bioavailability under physiological conditions. It should be noted that molecular docking in this study was primarily employed as a hypothesis-generating tool to explore potential interactions between the identified peptides and inflammation-related targets, rather than to establish definitive binding specificity. Given the complex nature of peptide–protein interactions, it is possible that these peptides may exert their anti-inflammatory effects through multiple molecular targets and signaling pathways. Therefore, future studies should extend the current work by investigating additional inflammation-related targets and pathways, as well as performing comparative docking and experimental validation to more comprehensively elucidate the mechanisms underlying their bioactivity.

## Figures and Tables

**Table 1:** Physicochemical properties, bioactivity scores, toxicity prediction, bioavailability and molecular docking analysis of candidate peptides with anti-inflammatory potential.

Sequence	DGIPGMPG	PGFHVPPP	GPKGYPGP
Mass (Da)	742.84	847.09	771.86
Isoelectric point (pI)	5.7	8.32	9.79
Net charge at pH 7.0	0	0	+1
Hydrophobicity (kcal/mol)	9.10	9.77	13.86
Peptide Ranker	0.80	0.86	0.80
ToxinPred	Non-Toxin	Non-Toxin	Non-Toxin
CPPpred	0.11	0.08	0.12
Anti-inflammatory score	0.57	0.54	0.71
Estimated ΔG with 3FXI (kcal/mol)	−8.4	−8.3	−8.3

**Table 2:** Predicted hydrogen bonds and hydrophobic contacts between candidate peptides and the TLR4/MD-2/LPS complex in the top-ranked docking pose.

Peptide	Interaction Type	Hydrogen Bonding and Hydrophobic Residues of TLR4/MD-2/LPS Complex
DGIPGMPG	hydrogen bond	Arg460
	hydrophobicity	His458, Ile466, Asn481, Leu434, Phe443, Phe429, Ser416, Thr413, Phe418, Gly389, Phe448, Lys435, Glu439, Ile454
PGFHVPPP	hydrogen bond	His426, Phe429
	hydrophobicity	Asp405, Phe408, Leu476, Ser407, Thr457, Phe467, Gln436, Val461, Met478, Leu452, Lys477, Leu427
GPKGYPGP	hydrogen bond	Leu476
	hydrophobicity	Asn526, Glu474, Asp453, Leu453, Leu427, Cys391, Phe418, Ile412, Cys390, Leu406, Thr413, Val461, Met414, Ile466, Thr457, Thr459, Asn481, Gly480, Leu503, Ser504

## Data Availability

The original contributions presented in this study are included in the article/Supplementary Material. Further inquiries can be directed to the corresponding author.

## Supplementary Materials

The following supporting information can be downloaded at: https://www.mdpi.com/article/10.3390/md24060192/s1, Figure S1: Concentration-dependent inhibition of LPS-induced NO production by JP-FC in RAW264.7 macrophages. Apparent IC_50_ values were estimated based on NO inhibition rates at different concentrations of JP-FC (25–400 μg/mL). Data are presented as mean ± SD (*n* = 3); Figure S2: Base peak chromatogram (BPC) of JP-FC obtained by LC-MS/MS analysis for peptide profiling; Figure S3: HPLC purity profile and mass spectrometry characterization of the synthesized peptide DGIPGMPG; Figure S4: HPLC purity profile and mass spectrometry characterization of the synthesized peptide PGFHVPPP; Figure S5: HPLC purity profile and mass spectrometry characterization of the synthesized peptide GPKGYPGP; Table S1: HPLC purity analysis of the synthesized anti-inflammatory peptides.

## References

[1] Zhao K., Zeng Z., He Y., Zhao R., Niu J., Sun H., Li S., Dong J., Jing Z., Zhou J. (2024). Recent Advances in Targeted Therapy for Inflammatory Vascular Diseases. J. Control. Release.

[2] Satapathy T., Patel N., Sahu P., Satapathy A. (2025). Decoding Inflammatory Signaling Networks: From Molecular Mechanisms to Therapeutic Targets. Adv. Biomark. Sci. Technol..

[3] Chen L., Deng H., Cui H., Fang J., Zuo Z., Deng J., Li Y., Wang X., Zhao L. (2018). Inflammatory Responses and Inflammation-Associated Diseases in Organs. Oncotarget.

[4] Wang B., Gong X., Wan J., Zhang L., Zhang Z., Li H., Min S. (2011). Resolvin D1 Protects Mice from LPS-Induced Acute Lung Injury. Pulm. Pharmacol. Ther..

[5] Jomova K., Alomar S.Y., Valko R., Liska J., Nepovimova E., Kuca K., Valko M. (2025). Flavonoids and Their Role in Oxidative Stress, Inflammation, and Human Diseases. Chem.-Biol. Interact..

[6] Bernal-Bello D., Frutos-Pérez B., Duarte-Millán M.Á., Toledano-Macías M., Jaenes-Barrios B., Morales-Ortega A. (2025). Cancer Risk in Autoimmune and Immune-Mediated Diseases: A Narrative Review for Practising Clinicians. J. Clin. Med..

[7] Shafiq A., Shah H., Khan A., Khan S., Akram F., Shafique M., Sattar F., Hanif M.F., Hashmat M.S., Madni A. (2025). Folate Receptor-Mediated Targeting Strategies for Potential Management & Treatment of Rheumatoid Arthritis. J. Drug Deliv. Sci. Technol..

[8] Tawfik A.G., Gomez-Lumbreras A., Del Fiol G., Kawamoto K., Trinkley K.E., Reese T., Jones A., Malone D.C. (2026). Nonsteroidal Anti-Inflammatory Drugs and Risk of Gastrointestinal Bleeding: A Systematic Review and Meta-Analysis. Clin. Pharma. Ther..

[9] Chapa-Villarreal F.A., Stephens M., Pavlicin R., Beussman M., Peppas N.A. (2024). Therapeutic Delivery Systems for Rheumatoid Arthritis Based on Hydrogel Carriers. Adv. Drug Deliv. Rev..

[10] Carter G.T., Duong V., Ho S., Ngo K.C., Greer C.L., Weeks D.L. (2014). Side Effects of Commonly Prescribed Analgesic Medications. Phys. Med. Rehabil. Clin. N. Am..

[11] Roubille C., Martel-Pelletier J., Davy J.M., Haraoui B., Pelletier J.P. (2013). Cardiovascular Adverse Effects of Anti-Inflammatory Drugs. Anti-Inflamm. Anti-Allergy Agents Med. Chem..

[12] Wu Z., Zhang T., Ma X., Guo S., Zhou Q., Zahoor A., Deng G. (2023). Recent Advances in Anti-Inflammatory Active Components and Action Mechanisms of Natural Medicines. Inflammopharmacology.

[13] Rao P.P. (2025). Natural Products and Nutraceuticals in the Management of Chronic Inflammatory Diseases: Efficacy, Mechanisms, and Comparative Insights. Inflammopharmacology.

[14] Lafarga T., Hayes M. (2014). Bioactive Peptides from Meat Muscle and By-Products: Generation, Functionality and Application as Functional Ingredients. Meat Sci..

[15] Chakrabarti S., Jahandideh F., Wu J. (2014). Food-Derived Bioactive Peptides on Inflammation and Oxidative Stress. BioMed Res. Int..

[16] Jiehui Z., Liuliu M., Haihong X., Yang G., Yingkai J., Lun Z., An Li D.X., Dongsheng Z., Shaohui Z. (2014). Immunomodulating Effects of Casein-Derived Peptides QEPVL and QEPV on Lymphocytes in Vitro and in Vivo. Food Funct..

[17] Zhao L., Wang X., Zhang X.L., Xie Q.F. (2016). Purification and Identification of Anti-Inflammatory Peptides Derived from Simulated Gastrointestinal Digests of Velvet Antler Protein (*Cervus elaphus* Linnaeus). J. Food Drug Anal..

[18] Gunasekara D.M.N.M., Wijerathne H.D.T.U., Wang L., Kim H.-S., Sanjeewa K.K.A. (2025). Marine Bioactive Peptides in the Regulation of Inflammatory Responses: Current Trends and Future Directions. Proteomes.

[19] Fu Y., Kim H., Lee D.S., Han A., Heine H., Zamyatina A., Kim H.M. (2025). Structural Insight into TLR4/MD-2 Activation by Synthetic LPS Mimetics with Distinct Binding Modes. Nat. Commun..

[20] Hering M., Madi A., Sandhoff R., Ma S., Wu J., Mieg A., Richter K., Mohr K., Knabe N., Stichling D. (2024). Sphinganine Recruits TLR4 Adaptors in Macrophages and Promotes Inflammation in Murine Models of Sepsis and Melanoma. Nat. Commun..

[21] Wei J., Zhang Y., Li H., Wang F., Yao S. (2023). Toll-like Receptor 4: A Potential Therapeutic Target for Multiple Human Diseases. Biomed. Pharmacother..

[22] Tandon A., Pathak M., Harioudh M.K., Ahmad S., Sayeed M., Afshan T., Siddiqi M.I., Mitra K., Bhattacharya S.M., Ghosh J.K. (2018). A TLR4-Derived Non-Cytotoxic, Self-Assembling Peptide Functions as a Vaccine Adjuvant in Mice. J. Biol. Chem..

[23] Wu J., Kong J., Laws E.A., Liu X., Wang C., Chen J., Chen M., Yao Q., Wang Y., Zhen Y. (2023). The Link Between Marine Thermal Discharges and *Nemopilema nomurai* Blooms Around Nuclear Power Plants. Ecosyst. Health Sustain..

[24] Purcell J., Uye S., Lo W. (2007). Anthropogenic Causes of Jellyfish Blooms and Their Direct Consequences for Humans: A Review. Mar. Ecol. Prog. Ser..

[25] Costa R., Capillo G., Albergamo A., Li Volsi R., Bartolomeo G., Bua G., Ferracane A., Savoca S., Gervasi T., Rando R. (2019). A Multi-Screening Evaluation of the Nutritional and Nutraceutical Potential of the Mediterranean Jellyfish *Pelagia noctiluca*. Mar. Drugs.

[26] Yu H., Li R., Liu S., Xing R., Chen X., Li P. (2014). Amino Acid Composition and Nutritional Quality of Gonad from Jellyfish *Rhopilema esculentum*. Biomed. Prev. Nutr..

[27] Nishimoto S., Goto Y., Morishige H., Shiraishi R., Doi M., Akiyama K., Yamauchi S., Sugahara T. (2008). Mode of Action of the Immunostimulatory Effect of Collagen from Jellyfish. Biosci. Biotechnol. Biochem..

[28] De Domenico S., De Rinaldis G., Paulmery M., Piraino S., Leone A. (2019). Barrel Jellyfish (*Rhizostoma pulmo*) as Source of Antioxidant Peptides. Mar. Drugs.

[29] Lv Z., Zhang C., Song W., Chen Q., Wang Y. (2022). Jellyfish Collagen Hydrolysate Alleviates Inflammation and Oxidative Stress and Improves Gut Microbe Composition in High-Fat Diet-Fed Mice. Mediat. Inflamm..

[30] Prommasith P., Surayot U., Autsavapromporn N., Rod-in W., Rachtanapun P., Wangtueai S. (2024). Immunomodulatory, Anticancer, and Antioxidative Activities of Bioactive Peptide Fractions from Enzymatically Hydrolyzed White Jellyfish (*Lobonema smithii*). Foods.

[31] Zhuang Y., Sun L., Zhang Y., Liu G. (2012). Antihypertensive Effect of Long-Term Oral Administration of Jellyfish (*Rhopilema esculentum*) Collagen Peptides on Renovascular Hypertension. Mar. Drugs.

[32] Teng L., Wang X., Yu H., Li R., Geng H., Xing R., Liu S., Li P. (2023). Jellyfish Peptide as an Alternative Source of Antioxidant. Antioxidants.

[33] Guha S., Majumder K. (2019). Structural-Features of Food-Derived Bioactive Peptides with Anti-Inflammatory Activity: A Brief Review. J. Food Biochem..

[34] Liu W., Chen X., Li H., Zhang J., An J., Liu X. (2022). Anti-Inflammatory Function of Plant-Derived Bioactive Peptides: A Review. Foods.

[35] Park B.S., Song D.H., Kim H.M., Choi B.S., Lee H., Lee J.O. (2009). The Structural Basis of Lipopolysaccharide Recognition by the TLR4–MD-2 Complex. Nature.

[36] Zhang Y., Liang X., Bao X., Xiao W., Chen G. (2022). Toll-like Receptor 4 (TLR4) Inhibitors: Current Research and Prospective. Eur. J. Med. Chem..

[37] Akbarian M., Khani A., Eghbalpour S., Uversky V.N. (2022). Bioactive Peptides: Synthesis, Sources, Applications, and Proposed Mechanisms of Action. Int. J. Mol. Sci..

[38] Zhang H., Kovacs-Nolan J., Kodera T., Eto Y., Mine Y. (2015). γ-Glutamyl Cysteine and γ-Glutamyl Valine Inhibit TNF-α Signaling in Intestinal Epithelial Cells and Reduce Inflammation in a Mouse Model of Colitis via Allosteric Activation of the Calcium-Sensing Receptor. Biochim. Biophys. Acta (BBA)-Mol. Basis Dis..

[39] Cheng M.L., Wang H.C., Hsu K.C., Hwang J.S. (2015). Anti-Inflammatory Peptides from Enzymatic Hydrolysates of Tuna Cooking Juice. Food Agric. Immunol..

[40] Montoya-Rodríguez A., De Mejía E.G., Dia V.P., Reyes-Moreno C., Milán-Carrillo J. (2014). Extrusion Improved the Anti-inflammatory Effect of Amaranth (*Amaranthus hypochondriacus*) Hydrolysates in LPS-induced Human THP-1 Macrophage-like and Mouse RAW 264.7 Macrophages by Preventing Activation of NF-κ B Signaling. Mol. Nutr. Food Res..

[41] Rivera-Jiménez J., Berraquero-García C., Pérez-Gálvez R., García-Moreno P.J., Espejo-Carpio F.J., Guadix A., Guadix E.M. (2022). Peptides and Protein Hydrolysates Exhibiting Anti-Inflammatory Activity: Sources, Structural Features and Modulation Mechanisms. Food Funct..

[42] Rodrigues C.H.M., Garg A., Keizer D., Pires D.E.V., Ascher D.B. (2022). CSM-peptides: A Computational Approach to Rapid Identification of Therapeutic Peptides. Protein Sci..

[43] Shen W., Wang X., Liu X., Li R., Liu S., Xing R., Yu H. (2026). Novel Antioxidant Peptides from Jellyfish (*Nemopilema nomurai*) Hydrolysate: Identification, Activity, and Potential Mechanisms. Food Chem..

[44] Li M., Dong L., Du H., Bao Z., Lin S. (2021). Potential Mechanisms Underlying the Protective Effects of *Tricholoma matsutake* Singer Peptides against LPS-Induced Inflammation in RAW264.7 Macrophages. Food Chem..

[45] Mooney C., Haslam N.J., Pollastri G., Shields D.C. (2012). Towards the Improved Discovery and Design of Functional Peptides: Common Features of Diverse Classes Permit Generalized Prediction of Bioactivity. PLoS ONE.

[46] Gupta S., Kapoor P., Chaudhary K., Gautam A., Kumar R., Open Source Drug Discovery Consortium, Raghava G.P.S. (2013). In Silico Approach for Predicting Toxicity of Peptides and Proteins. PLoS ONE.

[47] Holton T.A., Pollastri G., Shields D.C., Mooney C. (2013). CPPpred: Prediction of Cell Penetrating Peptides. Bioinformatics.

[48] Minkiewicz P., Iwaniak A., Darewicz M. (2019). BIOPEP-UWM Database of Bioactive Peptides: Current Opportunities. Int. J. Mol. Sci..

