# Supplementary material for: Identification of Novel Anti-Inflammatory Peptides from Jellyfish Nemopilema nomurai Enzymatic Hydrolysate: An Integrated In Silico Analysis and Cellular Evaluation
Source: Mar Drugs. 2026 May 28;24(6):192. doi: 10.3390/md24060192 (PMC13301869; doi:10.3390/md24060192)
Supplement: Supplementary file 1 [file marinedrugs-24-00192-s001.zip › marinedrugs-4293858-supplementary.pdf]

To further evaluate the concentration-dependent anti-inflammatory activity of JP-FC, additional concentration gradients (25–400 μg/mL) were analyzed using the LPS-stimulated RAW264.7 macrophage model. As shown in Figure S1, JP-FC exhibited a concentration-dependent inhibitory effect on NO production. The inhibitory activity gradually increased with increasing concentration and reached the highest level at 400 μg/mL. Based on the NO inhibition rates, the apparent IC_{50} value of JP-FC was estimated to be 235.6 μg/mL, supporting the potential anti-inflammatory activity of JP-FC in this cellular model.

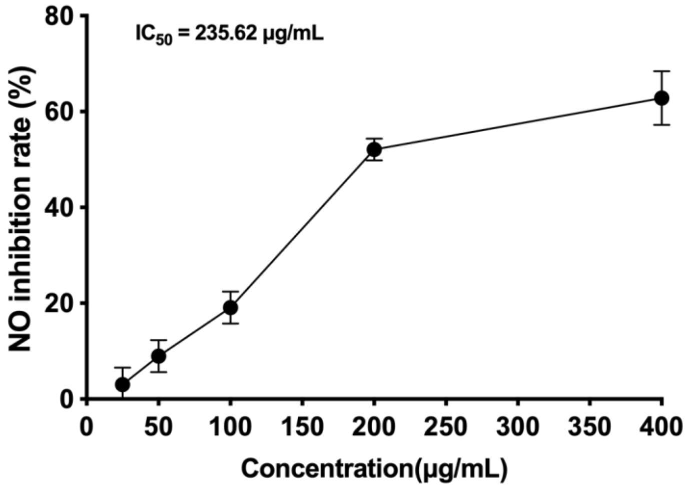

Figure S1. Concentration-dependent inhibition of LPS-induced NO production by JP-FC in RAW264.7 macrophages. Apparent IC_{50} values were estimated based on NO inhibition rates at different concentrations of JP-FC (25–400 μg/mL). Data are presented as mean ± SD (n = 3).

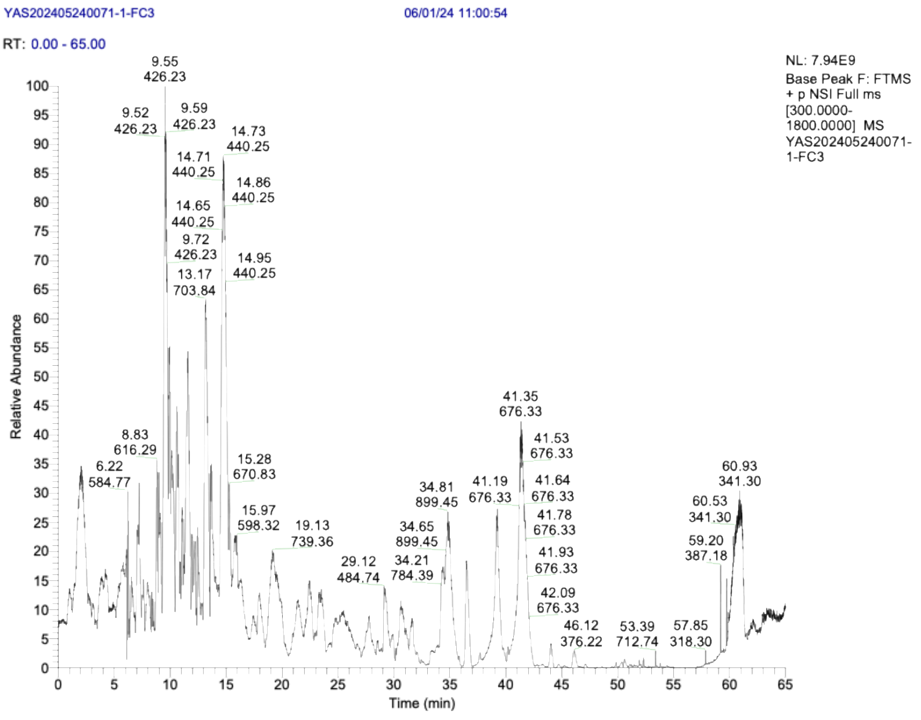

Figure S2. Base peak chromatogram (BPC) of JP-FC obtained by LC-MS/MS analysis for peptide

profiling.

肽谷生物 www.tgpeptide.com 南京肽谷生物科技有限公司

Certificate of Analysis

质量分析报告(COA)

Product Name（产品名称）: TG-LG-24503

Lot No（批号）: TP-20083820

Peptide Sequence（肽序）: DGIPGMPG

Molecular Weight（分子量）: 742.84

Weight（重量）: 4mg

Storage（储存）: -4℃or -20℃

Solubility（溶解性）： Normal

检测项目<br>Test Items	标准规定<br>Specification	检测结果<br>Results
Appearances 外观	------	powder
Purity (HPLC)纯度	>98%	98.80%
Mass 质谱	see attached MS Analysis Report	

***Note: this product is intended for research use only; not for diagnostic or human use.**

***Important: Stable at 2-8°C, but should be kept at -20°C for long term storage, preferably desiccated.**

Quality Assurance By:______________ Date: 2025.08.26

Quality Control Department

南京肽谷生物科技有限公司
地址：南京市江北新区天圣路 22 号 F 栋 邮箱：sales@tgpeptide.com
邮编: 210000

HPLC Analysis Report

Sample ID: TG-LG-24503

Sequence: DGIPGMPG

Column: 4.6*250mm, kromasil C18-5

Solvent A: 0.1%Trifluoroacetic in 100% Acetonirile

Solvent B: 0.1%Trifluoroacetic in 100% Water

Gradient:		A	B
	0.01min	5%	95%
	25.0min	50%	50%
	30min	90%	10%

Flow rate: 1.0ml/min Wavelength: 214nm Volume: 20ul

自动标尺色谱图

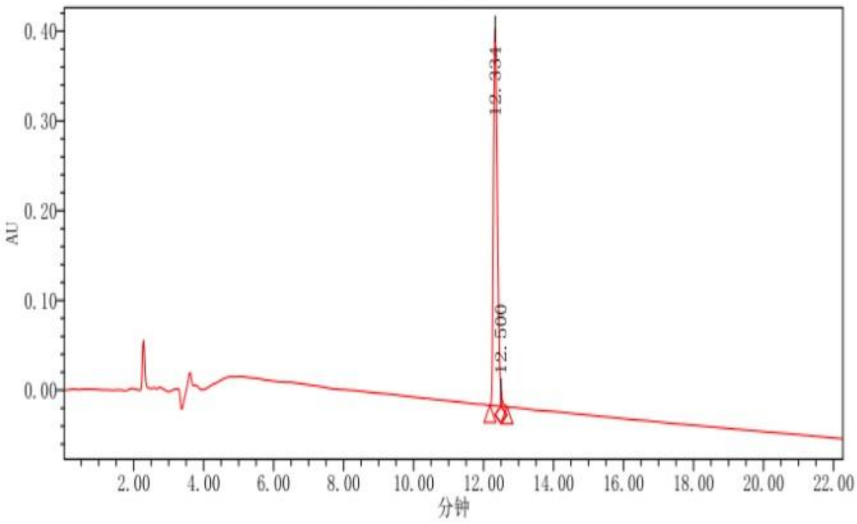

峰结果

	保留时间 (分钟)	面积 (微伏*秒)	高度 (微伏)	% 面积
1	12.334	3169368	419242	98.80
2	12.500	38511	16305	1.20

南京肽谷生物科技有限公司
地址：南京市江北新区天圣路 22 号 F 栋 邮箱：sales@tgpeptide.com
邮编: 210000

肽谷生物

www.tgpeptide.com 南京肽谷生物科技有限公司

MS Analysis Report

Sample ID: TG-LG-24503

Expected MS: 742.84

Flow rate: 0.2ml/min Run Time: 1min

Buffer A: 0.1% HCOOH in water Buffer B: 0.1% HCOOH in Acetonirile

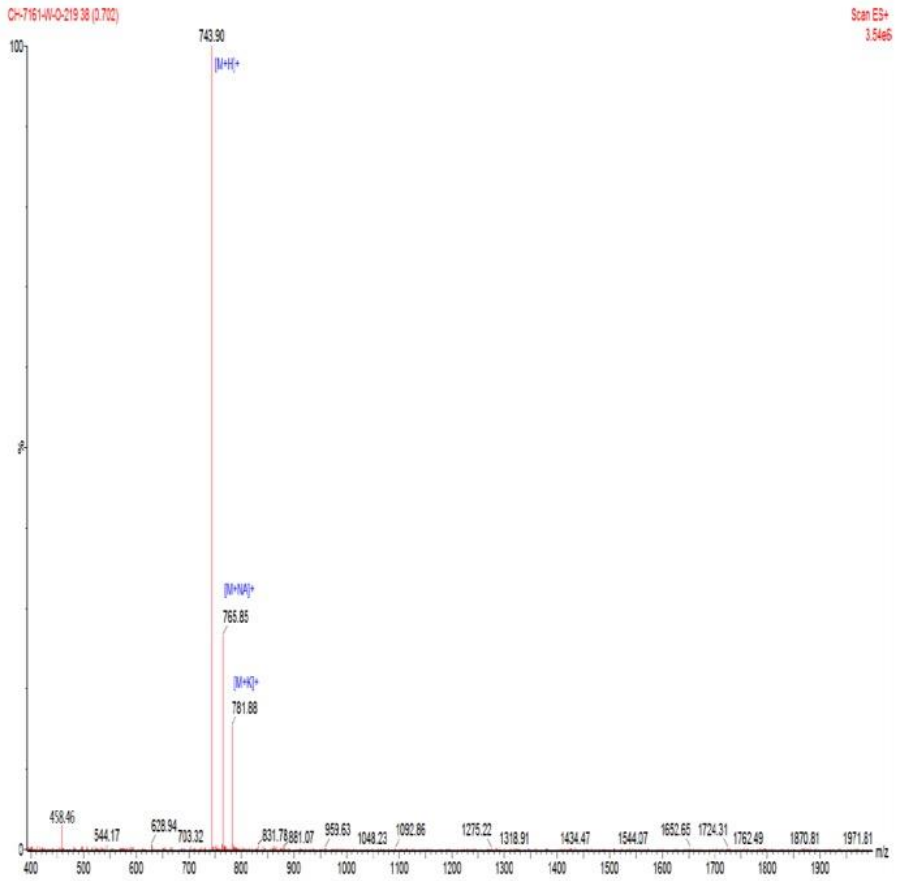

南京肽谷生物科技有限公司
地址：南京市江北新区天圣路 22 号 F 栋 邮箱：sales@tgpeptide.com
邮编: 210000

Figure S3. HPLC purity profile and mass spectrometry characterization of the synthesized peptide DGIPGMPG.

Certificate of Analysis

质量分析报告(COA)

Product Name（产品名称）: TG-LG-24505

Lot No（批号）: TP-20083822

Peptide Sequence（肽序）: PGFHVPPP

Molecular Weight（分子量）: 847.09

Weight（重量）: 4mg

Storage（储存）: -4°Cor -20℃

Solubility（溶解性）：Normal

检测项目<br>Test Items	标准规定<br>Specification	检测结果<br>Results
Appearances 外观	------	powder
Purity (HPLC)纯度	>98%	98.05%
Mass 质谱	see attached MS Analysis Report	

***Note: this product is intended for research use only; not for diagnostic or human use.**

***Important: Stable at 2-8°C, but should be kept at -20°C for long term storage, preferably desiccated.**

Quality Assurance By:____________ **Date: 2025.08.26**

Quality Control Department

南京肽谷生物科技有限公司
地址：南京市江北新区天圣路 22 号 F 栋 邮箱：sales@tgpeptide.com
邮编: 210000

HPLC Analysis Report

Sample ID: TG-LG-24505

Sequence: PGFHVPPP

Column: 4.6*250mm, kromasil C18-5

Solvent A: 0.1%Trifluoroacetic in 100% Acetonirile

Solvent B: 0.1%Trifluoroacetic in 100% Water

Gradient:		A	B
	0.01min	5%	95%
	25.0min	50%	50%
	30min	90%	10%

Flow rate: 1.0ml/min Wavelength: 214nm Volume: 20ul

自动标尺色谱图

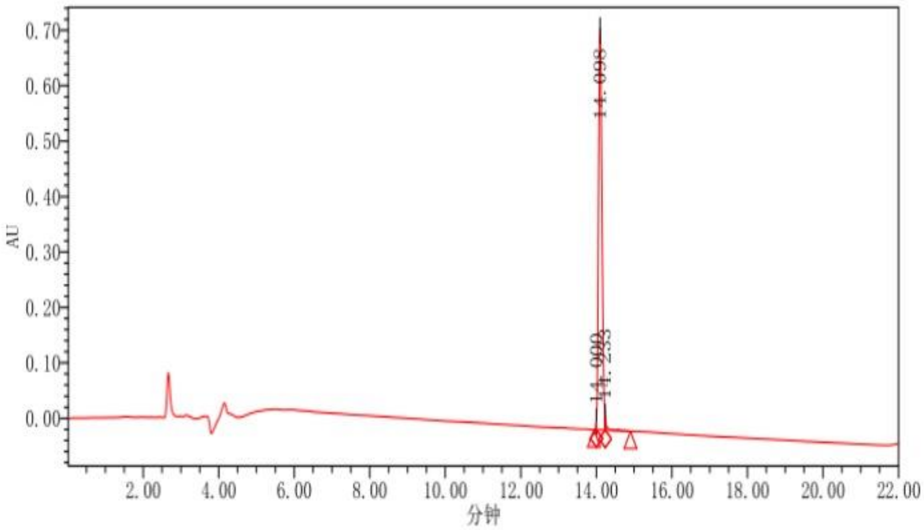

峰结果

	保留时间 (分钟)	面积 (微伏*秒)	高度 (微伏)	% 面积
1	14.000	10622	14504	0.23
2	14.098	4555661	721992	98.05
3	14.233	79990	23393	1.72

南京肽谷生物科技有限公司
地址：南京市江北新区天圣路 22 号 F 栋 邮箱：sales@tgpeptide.com
邮编: 210000

MS Analysis Report

Sample ID: TG-LG-24505

Expected MS: 847.09

Flow rate: 0.2ml/min Run Time: 1min

Buffer A: 0.1% HCOOH in water Buffer B: 0.1% HCOOH in Acetonirile

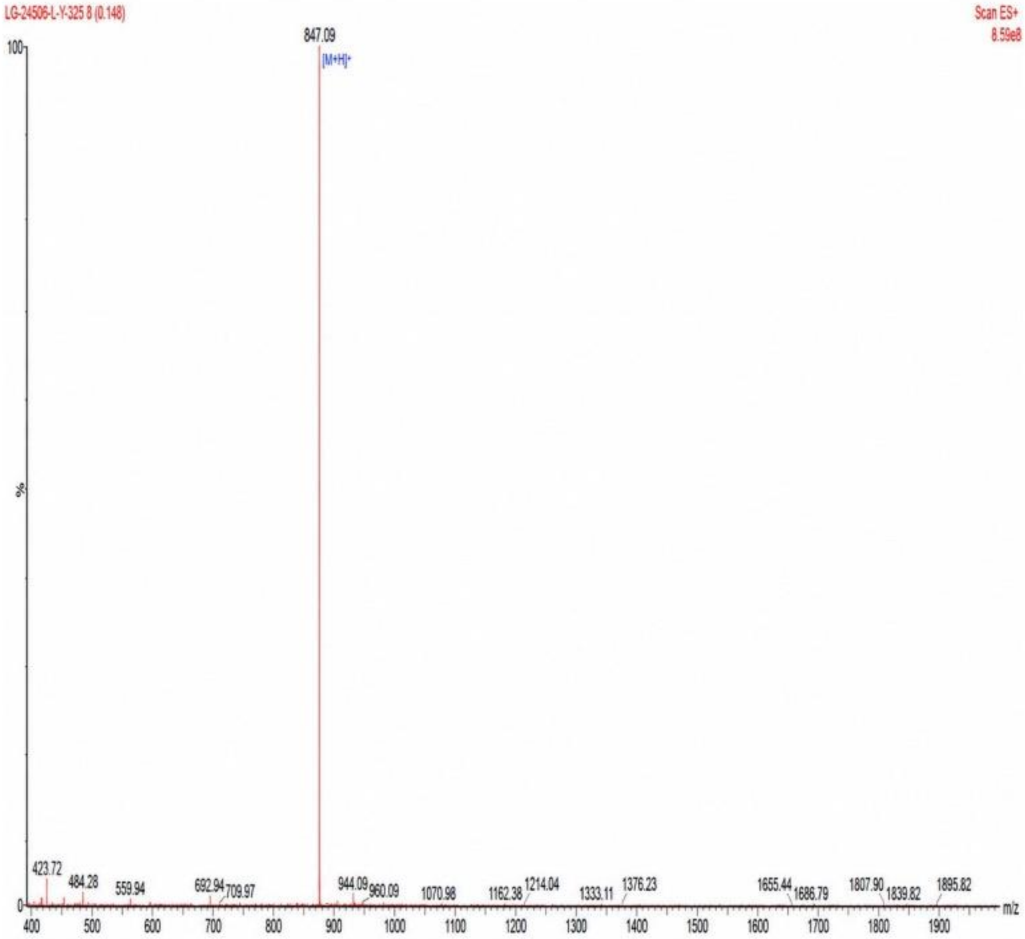

南京肽谷生物科技有限公司
地址：南京市江北新区天圣路 22 号 F 栋 邮箱：sales@tgpeptide.com
邮编: 210000

Figure S4. HPLC purity profile and mass spectrometry characterization of the synthesized peptide PGFHVPPP.

Certificate of Analysis

质量分析报告(COA)

Product Name（产品名称）: TG-LG-24504

Lot No（批号）: TP-20083821

Peptide Sequence（肽序）: GPKGYPGP

Molecular Weight（分子量）: 771.86

Weight（重量）: 4mg

Storage（储存）: -4℃or -20℃

Solubility（溶解性）： Normal

检测项目<br>Test Items	标准规定<br>Specification	检测结果<br>Results
Appearances 外观	------	powder
Purity (HPLC)纯度	>98%	98.72%
Mass 质谱	see attached MS Analysis Report	

***Note: this product is intended for research use only; not for diagnostic or human use.**

***Important: Stable at 2-8°C, but should be kept at -20℃ for long term storage, preferably desiccated.**

Quality Assurance By:____________ **Date: 2025.08.27**

Quality Control Department

HPLC Analysis Report

Sample ID: TG-LG-24504

Sequence: GPKGYPGP

Column: 4.6*250mm, kromasil C18-5

Solvent A: 0.1%Trifluoroacetic in 100% Acetonirile

Solvent B: 0.1%Trifluoroacetic in 100% Water

Gradient:		A	B
	0.01min	5%	95%
	25.0min	50%	50%
	30min	90%	10%

Flow rate: 1.0ml/min Wavelength: 214nm Volume: 20ul

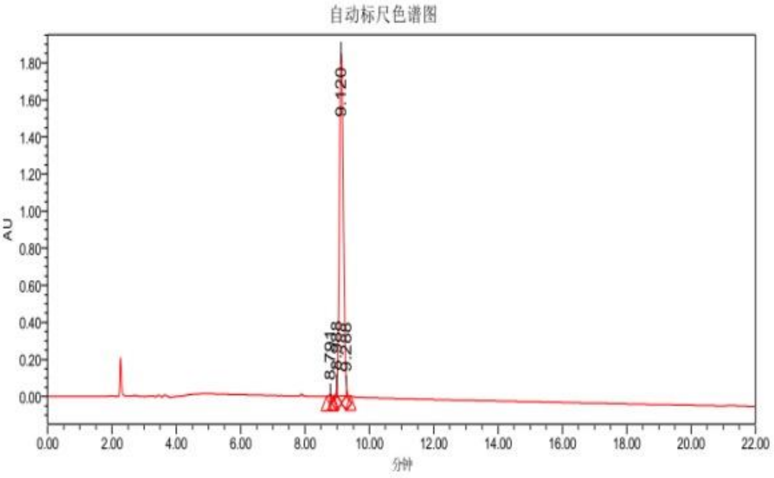

	保留时间 (分钟)	面积 (微伏*秒)	% 面积	高度 (微伏)
1	8.791	53650	0.33	11001
2	8.988	82337	0.51	60578
3	9.120	15826459	98.72	1852666
4	9.288	69303	0.43	51932

南京肽谷生物科技有限公司
地址：南京市江北新区天圣路 22 号 F 栋 邮箱：sales@tgpeptide.com
邮编: 210000

肽谷生物

www.tgpeptide.com 南京肽谷生物科技有限公司

MS Analysis Report

Sample ID: TG-LG-24504

Expected MS: 771.86

Flow rate: 0.2ml/min Run Time: 1min

Buffer A: 0.1% HCOOH in water Buffer B: 0.1% HCOOH in Acetonirile

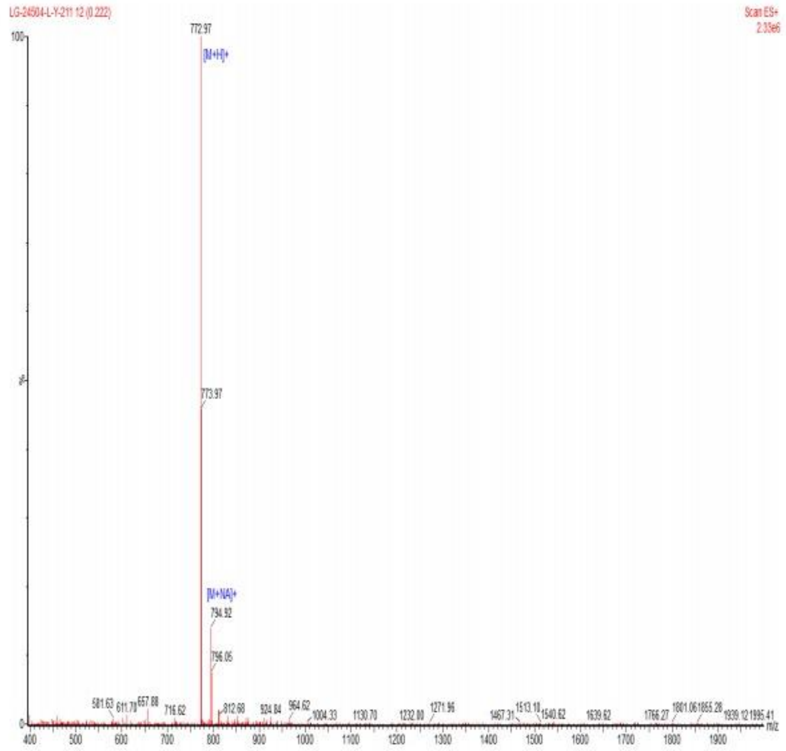

南京肽谷生物科技有限公司

地址：南京市江北新区天圣路 22 号 F 栋 邮箱：sales@tgpeptide.com

邮编: 210000

Figure S5. HPLC purity profile and mass spectrometry characterization of the synthesized peptide GPKGYPGP.

Supplementary Table S1. HPLC purity analysis of the synthesized anti-inflammatory peptides.

Peptide	Purity (%)	Observed m/z
DGIPGMPG	98.80	743.90
PGFHVPPP	98.05	847.09
GPKGYPGP	98.72	772.97
